# Optimization Design and Performance Evaluation of R1234yf Ejectors for Ejector-Based Refrigeration Systems

**DOI:** 10.3390/e24111632

**Published:** 2022-11-10

**Authors:** Meihong Yu, Chen Wang, Lei Wang, Hongxia Zhao

**Affiliations:** 1 School of Control Science and Engineering, Shandong University, Jinan 250061, China; 2 School of Energy and Power Engineering, Shandong University, Jinan 250061, China

**Keywords:** R1234yf ejector, performance improvement, ejector design, entrainment ratio, ejector refrigeration system

## Abstract

With the increasingly serious energy and environmental problems, the R1234yf ejector refrigeration system (ERS) shows great development potential in the refrigeration industry due to its simplicity, low maintenance costs and environmentally friendly nature. However, poor ejector performance has always been the main bottleneck for system applications. In order to overcome this problem, this paper proposes a design method for R1234yf ejectors based on the gas dynamic method and optimizes the geometrical parameters including the area ratio (AR) and nozzle exit position (NXP) to improve its performance through the control variable optimization algorithms. Based on the validated simulation model, the results show that the entrainment ratio increases initially and then decreases with the increase in AR and NXP, respectively; the AR has a significant effect on the shock wave position in the mixing chamber and the NXP can directly influence the expansion state of motive fluid; the ejector performance increases by about 17% over the initial entrainment ratio by the control variable optimization algorithms. This work can guide the R1234yf ejector design and promote the development of the ERS with environmentally friendly working fluids.

## 1. Introduction

With the rapid development of the global population and economy, the number of refrigeration systems has increased rapidly. The improvement of people’s comfort inevitably brings energy and environmental problems. In this scenario, the efficient cooling cycles and environmentally friendly refrigerants have been significant research hotspots in the refrigeration industry [1,2,3].

With the promulgation of Regulation No. 517/2014 (F-gas Regulation), the refrigerants with a high GWP will be phased out, such as R134A, R410A, R407C and so on [4]. Seeking alternatives to these working fluids has received extensive attention around the world. Among the alternatives, R1234yf is considered as the best choice because of its low GWP of 4, its ODP of 0 and its environmentally friendly nature. Minor et al. [5] explored the performance of the air-conditioning system with R1234yf, which was almost equal to that of the system with R134a. Jarall et al. [6] also gained similar results and pointed out that the system had the lower compression ratio compared with R134a. Del et al. [7] found that the refrigeration system components did not need to be modified when the R1234yf was used compared with CO_2_, which reduced the equipment investment. As a result, it can be concluded that the R1234yf is indeed an ideal alternative refrigerant for the refrigeration system.

The compressor is the main exergy-reduction component in conventional vapor compression refrigeration systems. The replacement of compressors with non-electricity-consuming components driven by waste heat or renewable energy is the main way to improve system overall performance. Currently, there are mainly three thermal-driven refrigeration systems: absorption, adsorption and ejector cooling systems. The adsorption cooling system has a difficult time achieving a higher refrigeration capacity and may cause system interruption when it works for a long time [8]. Furthermore, the absorption cooling system can achieve a high system efficiency compared with the other two systems, but it also has some drawbacks such as a high initial investment, system bulkiness and a high cooling temperature [9]. The ejector refrigeration system (ERS), as a novel cooling system, is a viable choice for vapor compression systems in order to decrease the exergy reduction of conventional compression refrigeration systems, in which the ejector is used to recollect expansion loss [10,11] or improve the fluid pressure [12,13] due to its simple system structure, low cost and moderate performance. Therefore, the ejector refrigeration system with R1234yf has been considered as an effective solution to energy shortages and environmental pollution in the refrigeration field. However, a low COP (Coefficient of Performance) is the main limitation for ERS with R1234yf compared with other thermal-driven cooling systems, especially the absorption refrigeration system, due to the poor performance of the ejector [9]. Therefore, most scholars have been improving the ejector performance because of the linear relationship between the system cooling capacity and the ejector entrainment ratio.

Ronanki et al. [14] investigated the system performance in a hybrid ERS system for automobiles, and the results showed that the system obtained the maximum entrainment ratio and COP when R1234yf is used compared with other refrigerants, with values of 0.45 and 0.3, respectively. Boumaraf et al. [15] investigated the R1234yf ejector performance using a more simple thermodynamic model in an ejector refrigeration system with two evaporators, and they stated that the COP of this system increases by about 17% in COP compared with R134a at high condensing temperatures. Fang et al. [16] evaluated the ejector performance numerically with R1234yf, R123ze(E) and R134a in a heat-driven ejector refrigeration system and found that R1234yf was a good substitution for R134a. Yan et al. [17] presented a compression heat pump cycle driven by solar energy with R134a and R1234yf; they pointed out that the heating exergy efficiency increased by about 52.8% compared with the conventional system. Expósito Carrillo et al. [18] proposed a novel ejector efficiency used in the thermodynamic methods to improve the cooling system performance with R1234yf, and the system COP can increase to 2.794 with an improvement of about 26% under the same conditions. Zhang et al. [19,20] presented a new ejector cooling system with a thermal pumping effect (ECSTPE) and double evacuation chambers and investigated the effects of refrigerants including R1234yf, R134a and R141b on system performance. They found that the R1234yf showed the best performance, with a COP of 0.49. From the above literature, we can conclude that the ERS with R1234yf does have the better performance compared with the ejector cooling system with conventional refrigerants. However, these studies mainly concentrate on the system modification using the thermodynamic methods to improve the performance of the ERS with R1234yf, and there are few papers about the optimizations of ejector performance, which is well established in the ERS with conventional refrigerants.

As for the optimization of ejector thermodynamic models with conventional working fluids in the ERS, Keenan [21] made two assumptions about the fluid mixing mode in the mixing chamber: constant-area and constant-pressure mixing. The latter theory is widely used in subsequent research, providing a theoretical basis for ejector mixing modeling. Huang et al. [22] presented a one-dimensional model to predict the ejector performance with R141b under the double-choking state and obtained the coupling relationship between the loss coefficient and the structural parameters. Zhu et al. [23] pointed out that there was a shock circle at the inlet of the constant-area mixing chamber. This theory simplifies the traditional one-dimensional model and can predict the ejector performance under the critical mode more accurately, making the theoretical model more consistent with the actual fluid flow characteristics inside the ejector. Ma et al. [24] innovatively put forward a one-dimensional modeling method to estimate the ejector performance with steam under the critical state by using steam sound velocity at the throat, and they analyzed the influence of ejector structural parameters and loss coefficients of different parts on ejector performance. To further analyze the non-equilibrium condensation phenomenon, Zhang et al. [25] proposed a modified model to predict the condensation phenomenon in a Moses and Stein nozzle and found that the modified model was more accurate than the original model in predicting the Wilson point position and its thermal parameters. Piotr et al. [26] thoroughly investigated the effects of four often-used condensation models on the condensation characteristics in moist air flows and recommended the most suitable model for moist air transonic flow. Zhang et al. [27] also studied the influence of impurities in steam ejectors on the non-equilibrium condensation phenomenon and pointed out that the process of steam condensation on salt particles had a significant impact on two-phase variables and flow parameters. The thermodynamic model of the ejector has been gradually improved with the efforts of scholars, but the influence of the ejector structural parameters on the ejector performance has not been considered. Expasito et al. [28] used a multi-objective optimization algorithm to optimize the ejector structural parameters with air based on CFD numerical simulation and pointed out that the nozzle throat and the diameter of the mixing chamber have the greatest impact on the ejector performance. Wang et al. [29] investigated the influence of nozzle structure on ejector performance with R134a and pointed out that the roughness of the nozzle diffuser and throat should be given more attention when designing ejectors. Wang et al. [30] put forward a mathematical model for designing the steam nozzle structure and pointed out that the change in the entrained fluid pressure can lead to the axial movement of the shock wave. Liu et al. [31] explored the influence of the steam ejector area ratio on the ejector efficiency of various components. They pointed out that the mixing chamber efficiency had the greatest impact on the ejector performance with the change in the area ratio. 

From the above literature reviews, we can see that the research into improving the ejector performance with conventional working fluids has matured. However, there are few reports on the thorough design and performance optimization methods of the R1234yf ejector in the ERS, which will hinder the performance improvement of the ejector and the ERS with R1234yf because the design approach is the cornerstone of optimizing ejector performance. Consequently, this paper proposes the detailed design method for the R1234yf ejector based on the aerodynamic method and optimizes the geometrical parameters to improve its performance through the control variable optimization algorithms, which will promote the development of the ERS with R1234yf.

## 2. R1234yf Ejector Design

### 2.1. The Ejector Refrigeration System

**Figure 1:**
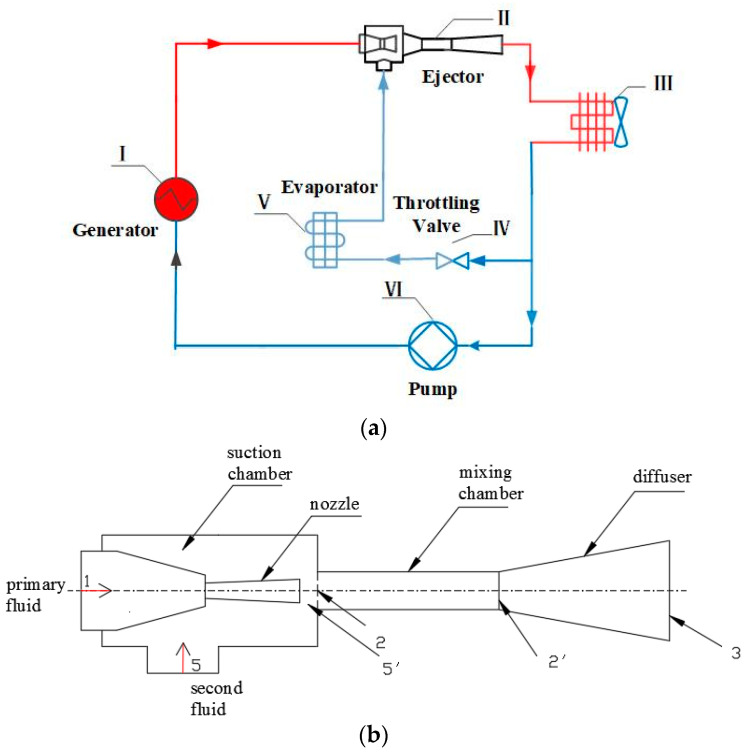
The ejector refrigeration system. (**a**) Diagram of the ejector-based refrigeration system. (**b**) Detailed structures of the ejector.

Figure 1a shows the diagram of the ejector-based refrigeration system, which mainly includes the generator, ejector, condenser, throttling valve, evaporator and pump. The primary fluid with a high temperature and pressure from the generator flows into the ejector, and the low-pressure vapor from the evaporator is entrained into the ejector due to the pressure difference between the two fluids. Then, the mixing fluid enters the condenser to release heat, and its state becomes liquid. Next, part of the liquid flows into the throttling valve, which is accompanied by a reduction in temperature and pressure, resulting in the production of gas–liquid two-phase fluid. Finally, the two-phase fluid absorbs heat from outside the evaporator, producing the cooling effect. At the same time, the fluid becomes gaseous and is entrained into the ejector. On the other hand, another part of the liquid from the condenser enters the generator after being pressurized by the mechanical pump to generate high-temperature and high-pressure fluid flowing into the ejector. The ejector is used to increase the pressure of fluid from the evaporator instead of the compressor, without energy consumption in the ERS.

Figure 1b depicts the detailed ejector structures, which consist of a suction chamber, nozzle, mixing chamber and diffuser. The working principle is that the motive flow from the generator flows into the converging and diverging nozzle, and its velocity increases to sonic initially at the throat due to the reduction in nozzle areas and then further accelerates to supersonic at the divergent section of the nozzle, creating a low-pressure region at the nozzle exit. The suction fluid is sucked into the ejector due to the pressure difference between the secondary fluid and the low-pressure region. Then, the two fluids begin to mix and exchange energy, mass and momentum in the mixing chamber through a series of turbulent phenomena such as shock waves and the development of the mixing layer, so the primary fluid velocity decreases and the secondary fluid velocity increases. As a result, the velocity of the two fluids tends to be the same at the end of the mixing chamber as the mixing process proceeds. Subsequently, the mixing fluid enters the diffuser, and its velocity reduces and its pressure increases because of the increase in the flow area. Therefore, the pressure potential energy of the motive flow converts into the suction fluid, resulting in the increase in suction fluid pressure. 

The entrainment ratio (μ) and pressure ratio (PR) are commonly used to evaluate the ejector performance in refrigeration systems. For a specified ejector used in the ejector refrigeration system, the high entrainment ratio is the objective parameter for the ejector performance optimization when the pressure ratio is satisfied, which means that more secondary flow is introduced, and a better cooling effect can be realized. The two parameters are defined as follows:

(1) $$\mu =\frac{m_{s}}{m_{p}}$$

where $m_{p}$ is the mass flow rate of the primary fluid, and $m_{s}$ is the mass flow rate of the secondary fluid.

(2) $$PR=\frac{P_{c}}{P_{s}}$$

where $P_{c}$ is the pressure of the mixing fluid at the ejector outlet, and $P_{s}$ is the pressure of the secondary fluid.

### 2.2. The R1234yf Ejector Design

It is difficult to design the satisfied ejector structure parameters due to the complex fluid flow field characteristics inside it, and scholars have been trying to present precise design methods for ejectors, among which the thermodynamic and gas dynamic design methods are universal. The gas dynamic design methods are more precise due to the presentation of formulas to determine the axial size of the ejector compared with the classical thermodynamic method; as a result, the structure parameters of the R1234yf ejector adopted in this paper are identified through this method.

To simplify the calculation process, the following assumptions are presented:

(1) The ejector is under steady conditions;
(2) The ejector wall is adiabatic.
(3) The velocity at the primary and secondary flow inlets and the ejector outlet is negligible compared with the supersonic velocity inside the ejector;
(4) The fluid and friction loss that occurs in the ejector is considered by the isentropic coefficients;

#### 2.2.1. Computational Model for Maximum Entrainment Ratio

The entrainment ratio calculated is expressed as follows:

(3) $$\mu =\frac{K_{1}\frac{a_{p*}}{a_{c*}}\lambda_{ps}-K_{3}\lambda_{c2^{\prime }}}{K_{4}\lambda_{c2^{\prime }}-K_{2}\frac{a_{h*}}{a_{c*}}\lambda_{s2}}$$

where the subscripts p, h and c represent motive fluid, entrained fluid and mixing fluid, respectively; a is the fluid critical velocity; λ is the converted-isentropic velocity; and K is defined as follows:

(4) $$K_{1}=\varphi_{1}\varphi_{2}\varphi_{3}$$

(5) $$K_{2}=\varphi_{2}\varphi_{3}\varphi_{4}$$

(6) $$K_{3}=1+\varphi_{3}\frac{a_{p*}}{a_{c*}}\frac{P_{c}}{P_{p}}\frac{\Pi_{c3}-\frac{P_{s}}{P_{c}}}{k_{p}\Pi_{p*}\lambda_{c3}q_{ps}}$$

(7) $$K_{4}=1+\varphi_{3}\frac{a_{p*}}{a_{c*}}\frac{P_{c}}{P_{s}}\frac{\Pi_{c3}-\Pi_{c5^{\prime }}}{k_{s}\Pi_{s*}\lambda_{c3}q_{s5^{\prime }}}$$

where $\varphi_{1}$,$\varphi_{2}$,$\varphi_{3}$,$\varphi_{4}$ represent the velocity coefficients of the fluid at the nozzle, mixing chamber inlet, mixing chamber outlet and diffuser, respectively. The specific values of these parameters are identified as: $\varphi_{1}=0.95$; $\varphi_{2}=0.975$; $\varphi_{3}=0.9$.

The fluid converted mass velocity at the mixing chamber inlet is expressed as:

(8) $$q_{s2}=\frac{\mu }{(1+\mu )\frac{a_{c*}}{a_{s*}}\frac{k_{s}}{k_{c}}\frac{\Pi_{h*}}{\Pi_{c*}}\frac{1}{q_{c3}}-\frac{a_{p*}}{a_{s*}}\frac{k_{s}}{k_{p}}\frac{\Pi_{s*}}{\Pi_{p*}}\frac{P_{s}}{P_{p}}\frac{1}{q_{ps}}}$$

where k is the fluid adiabatic exponent; Π is the relative pressure.

The fluid converted mass velocity at the mixing chamber outlet is calculated as:

(9) $$q_{c3}=\frac{\lambda_{c3}\varepsilon_{c3}}{\varepsilon_{c3}{}^{*}}$$

The ejector works under the second limit state when the secondary fluid reaches the critical speed at a certain section in the mixing chamber; at this time, the entrainment ratio is maximum and is defined as:

(10) $${\left(\mu_{np}\right)}_{2}=\frac{\frac{a_{c*}}{a_{h*}}\frac{k_{s}}{k_{c}}\frac{\Pi_{s*}}{\Pi_{c*}}\frac{P_{s}}{P_{c}}\frac{1}{q_{c3}}-\frac{a_{p*}}{a_{h*}}\frac{k_{s}}{k_{p}}\frac{\Pi_{s*}}{\Pi_{p*}}\frac{P_{s}}{P_{p}}\frac{1}{q_{ps}}}{1-\frac{a_{c*}}{a_{s*}}\frac{k_{s}}{k_{c}}\frac{\Pi_{s*}}{\Pi_{c*}}\frac{P_{s}}{P_{c}}\frac{1}{q_{c3}}}$$

**Figure 2:**
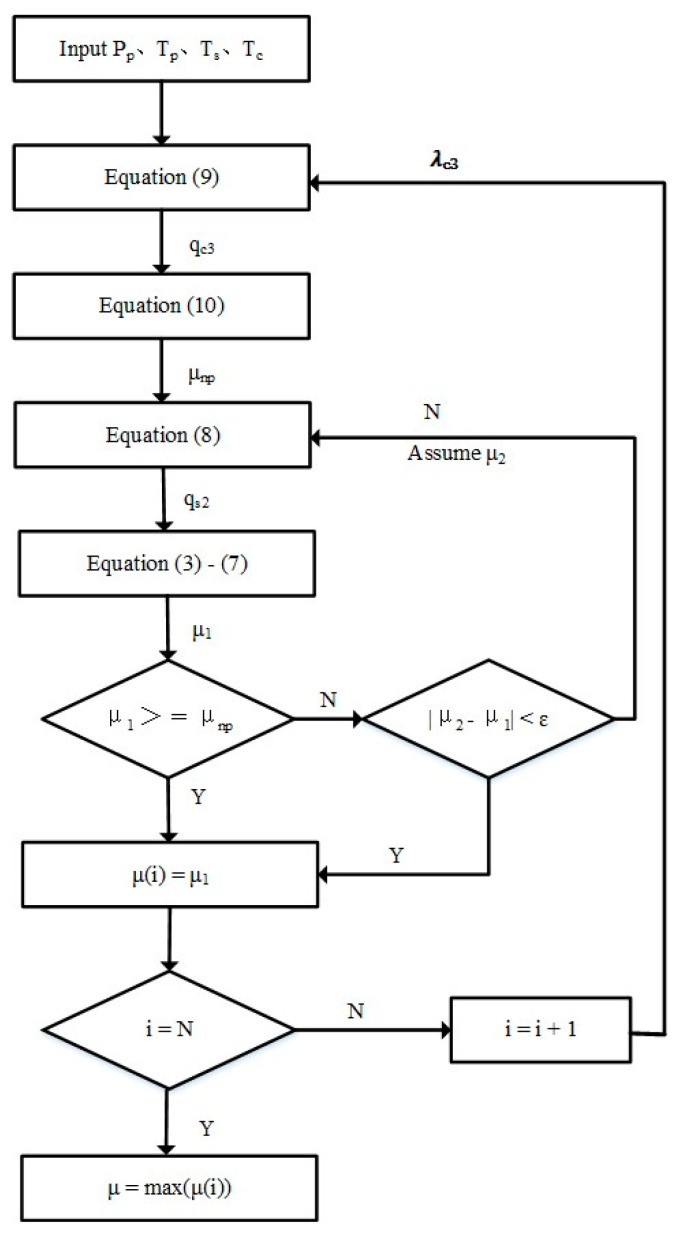
The calculation flow chart of the maximum entrainment ratio.

The maximum entrainment ratio is the optimization objective for ejector design with a satisfied pressure ratio, so it is considered a critical step for ejector design in computing the maximum entrainment ratio. Figure 2 shows the detailed calculation process.

#### 2.2.2. Computational Model for the Geometrical Parameters of the Ejector

The converging and diverging nozzle should be adopted to make the primary fluid fully expand when $\frac{P_{P}}{P_{h}}\;>\;\frac{1}{\Pi_{p*}}$, which is used in this work. The mass flow rate of the motive flow at the nozzle throat is defined as:

(11) $$m_{p}=f_{p*}\rho_{p*}a_{p*}$$

where $f_{p^{*}}$ is the nozzle throat area, $\rho_{p^{*}}$ is the fluid density at the nozzle throat and $a_{p^{*}}$ is the fluid critical velocity at the nozzle throat.

The diameter of the nozzle throat is:

(12) $$d_{p*}=\sqrt{\frac{4f_{p*}}{\pi }}$$

The nozzle outlet area is:

(13) $$f_{p2}=\frac{f_{p*}a_{p*}\rho_{p*}}{v_{p2}\rho_{p2}}=\frac{a_{p*}}{v_{p2}}\frac{\rho_{p*}}{\rho_{p}}\frac{\rho_{p}}{\rho_{p2}}=\frac{1}{q_{ph}}$$

where v is the fluid velocity.

The nozzle inlet area is expressed as:

(14) $$f_{p}=\frac{m_{p}}{\rho_{p}v_{p}}$$

The mixing chamber outlet area is defined as:

(15) $$f_{2^{\prime }}=\frac{f_{c*}}{q_{c3}}$$

where $f_{c^{*}}$ is the critical flow area of the mixing fluid.

The mixing chamber outlet diameter is:

(16) $$d_{2^{\prime }}=\sqrt{\frac{4f_{2^{\prime }}}{\pi }}$$

**Figure 3:**
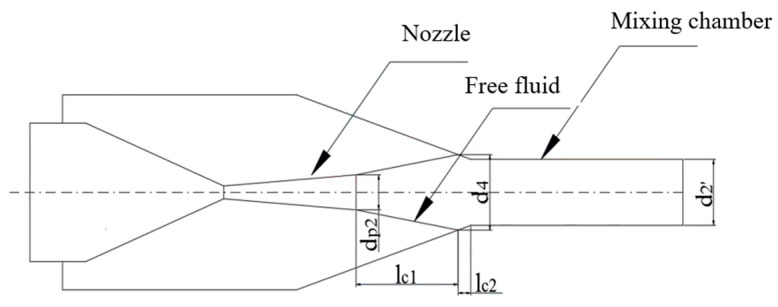
The free flow beam of the motive fluid.

The determination of the nozzle exit position mainly affects the expansion of motive fluid and then affects the mixing of two streams of fluid, which is considered to be an ideal value when the final section area of the primary fluid free flow beam coming out of the nozzle is equal to the mixing chamber inlet area. As a result, the free flow beam length of the motive fluid ($l_{c1}$) and the diameter of the free flow beam at this position ($d_{4}$) are two critical parameters in determining the nozzle exit position. Figure 3 is the diagram of the free flow beam of the motive fluid.

(1) The free flow beam length of the motive fluid $l_{c1}$

When the entrainment ratio is less than 0.5:

(17) $$l_{c1}=\left(\sqrt{0.083+0.76\mu }-0.29\right)\frac{d_{p2}}{2a}$$

when the entrainment ratio is higher than 0.5:

(18) $$l_{c1}=\frac{0.37+\mu }{4.4a}d_{p2}$$

where $d_{p2}$ is the nozzle outlet diameter, and a is the experimental constant.

(2) The fluid diameter at the end of the free flow beam ($d_{4}$)

When the entrainment ratio is less than 0.5:

(19) $$d_{4}=1.55\cdot d_{p2}\cdot (1+\mu )$$

when the entrainment ratio is higher than 0.5:

(20) $$d_{4}=3.4\cdot d_{p2}\cdot \sqrt{0.083+0.76\mu }$$

The nozzle exit position $l_{c}$ is $l_{c}=l_{c1}$ when the mixing chamber diameter is higher than the free flow beam diameter ($d_{2^{\prime }}\;>\;d_{4}$).

The nozzle exit position $l_{c}$ is $l_{c}=l_{c1}+l_{c2}$ when the mixing chamber diameter is less than the free flow beam diameter ($d_{2^{\prime }}\;<\;d_{4}$), where $l_{c2}$ is the distance from the end of the free flow beam to the mixing chamber inlet.

(21) $$l_{c2}=\frac{d_{4}-d_{2^{\prime }}}{2\tan\beta }$$

where β is the angle between the mixing chamber inlet and the axis of the ejector.

The optimal operating conditions of [32] are chosen as the ejector design parameters, and the other structural parameters are acquired according to the suggestion from [33]. Table 1 shows the main geometrical parameters.

### 2.3. The Control Variable Optimization Algorithms

**Figure 4:**
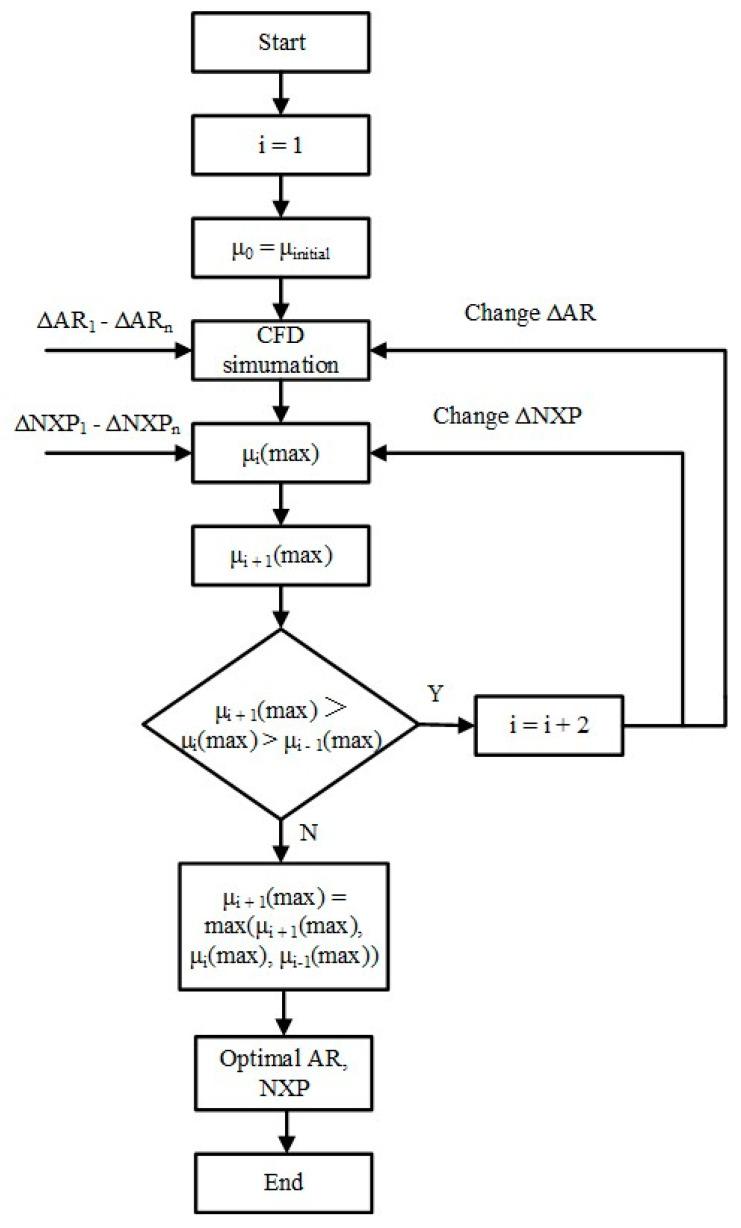
The control variable optimization algorithms.

We have to admit that the initial design cannot satisfy the optimal ejector performance because neither the theoretical nor the numerical simulation methods can accurately describe the fluid flow characteristics. Therefore, the structural optimization is an essential step in improving ejector performance for ejector design. This paper optimizes the ejector area ratio (AR) and nozzle exit position (NXP), which are two critical parameters related to fluid mixing and primary flow expansion, and finally obtains the optimal ejector performance. To ensure that the results are unaffected by other parameters, the simulation is carried out with fixed operation temperatures and geometry parameters, which are the same as the initial designed parameters. The control variable optimization algorithm is used to optimize the structural parameters in order to obtain optimum ejector performance, because these two geometrical parameters are coupled. Figure 4 shows the detailed optimization process.

## 3. Numerical Modeling and Validation

### 3.1. Governing Equations

The mass, momentum and energy conservation equations are the basis for solving the fluid flow characteristics inside the ejector, and they are expressed as follows based on above assumptions:

The continuity equation:

(22) $$\frac{\partial }{\partial x_{i}}(\rho u_{i})=0$$

The momentum equation:

(23) $$\frac{\partial }{\partial x_{i}}(\rho u_{i}u_{j})=-\frac{\partial P}{\partial x_{i}}+\frac{\partial \tau_{ij}}{\partial x_{j}}$$

The energy equation:

(24) $$\frac{\partial }{\partial x_{i}}(u_{i}(\rho E+P))=-\frac{\partial }{\partial x_{i}}(\lambda_{\mathrm{eff}}\frac{\partial T}{\partial x_{i}})+\frac{\partial }{\partial x_{i}}(u_{j}(\tau_{ij}))$$

(25) $$\tau_{ij}=\lambda_{eff}\left(\frac{\partial u_{i}}{\partial x_{j}}+\frac{\partial u_{j}}{\partial x_{i}}\right)-\frac{2}{3}\lambda_{eff}\frac{\partial u_{k}}{\partial x_{k}}\delta_{ij}$$

where i and j represent the fluid flow direction, and ρ, u, E, τ and λ represent the density, velocity, total energy, viscous stress and dynamic viscosity, respectively.

The turbulent model is used to close the fluid flow equations in the Reynolds-Averaged Navier–Stokes (RANS) numerical simulation methods, and the two-equation model is more widely used in engineering computation due to it being more theoretical when dealing with complex flow processes. Moreover, the prediction accuracy about fluid characteristics is higher for the k−ω model because it neglects the nonlinear attenuation function that is presented in the k−ε model. Giorgio et al. [34] reported that the SST k−ω shows the best results for fluid flow quantities inside the ejector compared with the RNG k−ε model, so this paper adopts the SST k−ω turbulent model to solve the fluid governing equations.

(26) $$v_{t}=\frac{a_{1}k}{\max(a_{1}\omega ,sF_{2})}$$

(27) $$F_{2}=\tan h(\Phi_{2}^{2})$$

(28) $$\Phi_{2}=\max\left[2\frac{\sqrt{k}}{\beta^{\prime }\omega y},\frac{500v}{y^{2\omega }}\right]$$

### 3.2. Solver and Numerical Settings

The Software Fluent 19.0 is used to complete numerical simulations, and the ejector works under a steady state. The 2D axisymmetric model was adopted to simplify the computation process. The turbulent SST k−ω was selected to solve the fluid flow characteristics inside the ejector. The “standard wall function” was used to calculate the turbulent quantities near the ejector wall, which is treated as a no-slip adiabatic boundary. The SIMPLE algorithm was utilized to deal with the pressure–velocity coupling equations, and the second-order upwind scheme was chosen for all discretization settings of the convection terms with the pressure-based solver. The motive flow and the entrained flow are R1234yf, and its density and other fluid thermal properties were obtained from NIST [35]. The motive flow and suction flow inlet boundaries were set as the pressure-inlet with pressure values of 2.8 MPa and 0.37 MPa, respectively. The outlet boundary was set as the pressure-outlet with the value of 0.7 MPa. For every computational case, the calculation cannot be considered as convergent until the residuals of all the numerical equations are lower than $10^{-6}$.

### 3.3. Meshing Technique

**Figure 5:**
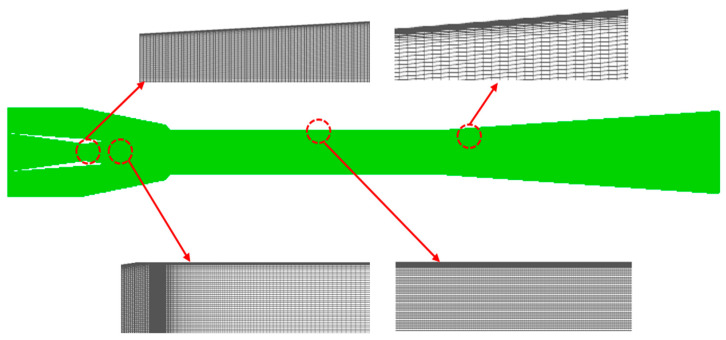
The detailed mesh technique.

**Figure 6:**
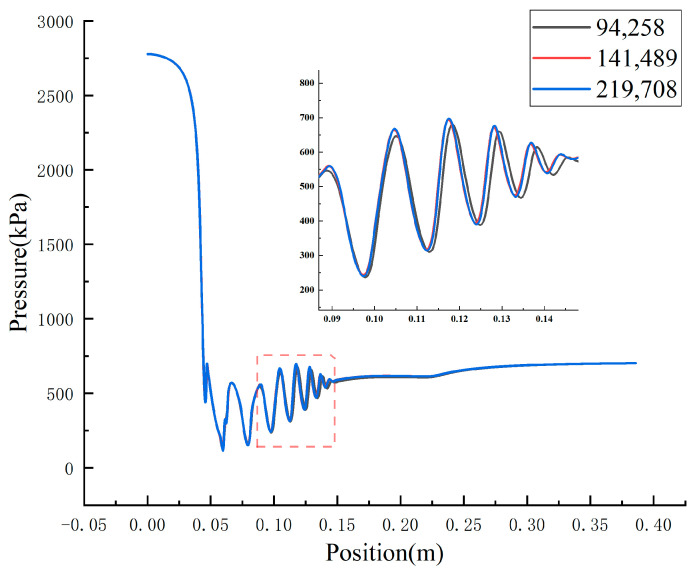
The pressure distribution under different mesh numbers.

Michael et al. [36] confirmed that the errors about ejector performance from the 2-D and 3-D models were in an acceptable range, so the 2-D axisymmetric model is adopted in this paper due to its low computational cost and high computational efficiency. As a result, the 2D structured meshes were produced by the commercial software ICEM (Integrated Computer Engineering and Manufacturing code), and the grids were refined at a region where the velocity gradient is high, such as the near-wall region and nozzle exit, to capture the more precise fluid flow phenomenon. Figure 5 shows the detailed mesh technique including the mesh refinement methods. The number of grids is essential to the calculation results during the numerical simulation. If the number of grids is too large, the error between the simulation and real results is so large that some important fluid characteristics are omitted. The computation cost will be increased when the number of grids is too small. As a result, grid independency verification is a necessary step to identify the mesh size and ensure the correctness of the calculation results. Figure 6 shows the pressure distribution along the ejector axis under different grid numbers. It can be seen that these profiles show the same change rule and nearly collapse in the axial direction of the ejector when the grid numbers increase from 94,258 to 219,708, meaning that the error is very small under different grid numbers.

The mixing chamber is an important component for the ejector where the primary fluid and secondary fluid exchange energy and the fluid velocity changes dramatically. As a result, the fluid field characteristic is very sensitive to grid numbers. In other words, it can be considered that the errors at every position on the axis are within the acceptable range when the change in flow characteristics at this region satisfies the error requirements under different grid densities. Table 2 shows the change in velocity and pressure at the central axis of the mixing chamber inlet under different grid numbers. It can be seen that all the errors are within 1%, which satisfies the computational accuracy requirements. As a result, the grid with 141,489 cells is used to predict the ejector performance considering the calculation cost and calculation time.

### 3.4. Model Validation

The results of the numerical model are generally considered as reliable if the errors of the entrainment ratio from simulations and experiments are acceptable [37]. The experimental results from Ref [22] are selected to judge the simulation methods adopted in this work. Table 3 shows the comparisons between the experiment and simulation results based on the same operation condition obtained from [22]. It can be seen that all the errors are within the acceptable range. The reason for the large error is that some assumptions have been made in solving the internal flow characteristics inside the ejector, resulting in the deviation between the simulation results and the complex flow occurring in the actual process. Nonetheless, the simulation model can also be utilized to predict the ejector performance with the acceptable error.

## 4. Results and Discussions

### 4.1. The Influence of the Geometric Parameters on the Ejector Performance

#### 4.1.1. The Influence of AR on Ejector Performance

**Figure 7:**
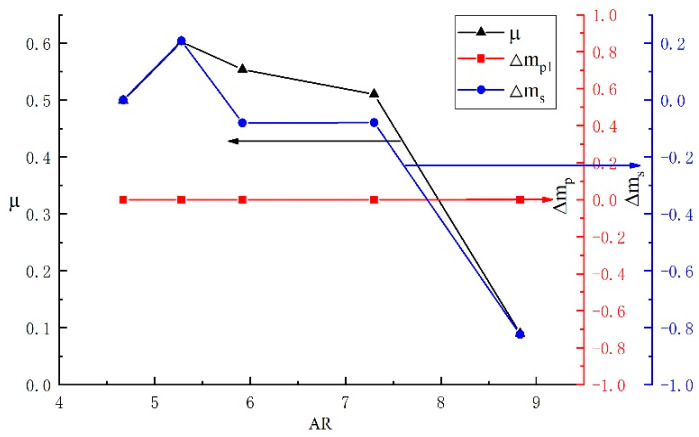
The effects of the area ratio on the entrainment ratio.

Figure 7 shows the effects of area ratios on the ejector entrainment ratio. It can be seen that the primary fluid mass flow rate remains constant, while both the growth rate of the secondary fluid mass flow rate and the entrainment ratio increase initially and then decrease with the increase in the area ratio. When the AR is 5.28, the secondary fluid mass flow growth rate reaches the maximum value; at this time, the secondary mass flow rate reaches the maximum value, resulting in the maximum entrainment ratio with a value of 0.602. Moreover, the growth rate of the secondary fluid mass flow rate is higher than 0 when the AR is lower than 5.28, and it becomes negative when the AR is higher than 5.28, which means that the backflow phenomenon occurs once the AR exceeds 5.28, leading to the decrease in the secondary fluid mass flow rate and entrainment ratio. The primary fluid mass flow rate is only related to the fluid characteristics in the nozzle, which remain constant when the AR varies; as a result, the increment rate of the primary fluid mass flow rate is always equal to 0 with the variation in AR. It can be concluded that the AR has a significant effect on the entrainment ratio and secondary mass flow rate and has little impact on the primary fluid mass flow rate.

**Figure 8:**
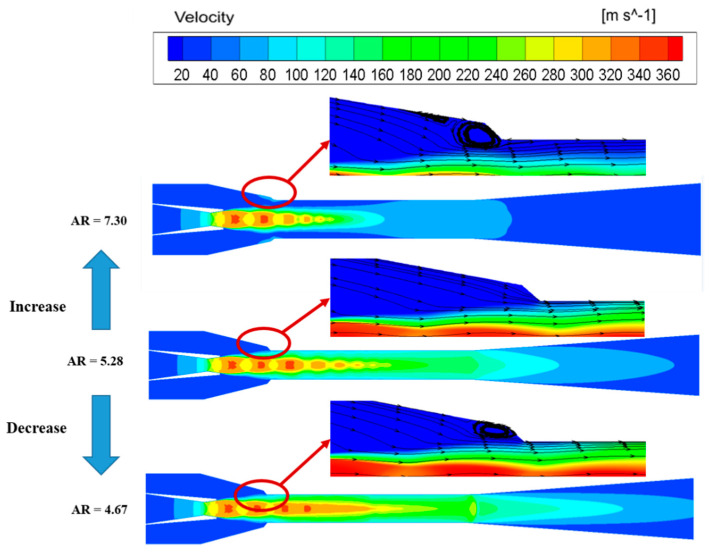
The flow field distribution inside the ejector under different ARs.

Figure 8 shows that there is a great difference in the flow field characteristics under different ARs. It can be seen that the shock wave disappears gradually at the end of the mixing chamber with the increase in the area ratio, and it can be concluded that the increase in AR can bring the shock wave to the mixing chamber inlet. When the AR is lower than the optimal area ratio, the kinetic energy that the primary fluid converts to the secondary fluid increases due to the decrease in the secondary fluid mass flow rate, so the mixed fluid velocity is still supersonic in the mixing chamber. When the fluid flows from the end of the mixing chamber to the diffuser, the shock wave appears, which increases the energy loss; therefore, the ejector has a poor performance. The shock wave disappears exactly at the end of the mixing chamber when the AR is 5.28, meaning that the primary fluid and the secondary fluid mix fully, which reduces the energy loss inside the ejector. As a result, the ejector has the best performance. Further increasing the area ratio causes too large of a flow space for the secondary fluid, so much of the secondary fluid cannot be entrained into the ejector, resulting in the disappearance of the shock wave and even the failure of the ejector.

Furthermore, the inappropriate AR will directly affect the flow area of the secondary flow, which in turn will cause the presentence of the backflow phenomenon. Figure 8 also shows the velocity streamline distribution inside the mixing chamber. It can be found that the backflow areas decrease initially and then increase inside the ejector with the increase in the AR. Moreover, the backflow phenomenon mainly concentrates on the mixing chamber inlet, indicating that the change in the area ratio mainly effects the mixing process of two fluids. When the AR is 4.67, the mixing chamber diameter is so small that the primary fluid fills the entire mixing chamber, and there is not enough space for the secondary fluid entrained into the ejector, which results in the fluid backflow. When the AR is at the optimal value, the mixing chamber diameter is appropriate for the mixing fluid, and the entrained secondary fluid can flow into the mixing chamber; as a result, there is no backflow phenomenon under this condition. The backflow phenomenon occurs again with the increase in the area ratio. In this event, the secondary fluid seems to flow into the large space, and the shear stress between the motive fluid and the entrained fluid is small. As a result, part of the fluid flows from the mixing chamber to the suction chamber because of the pressure difference between the inside and the outside of the ejector. In summary, the optimal AR of the ejector can eliminate the backflow phenomenon and reduce the shock wave loss inside the ejector.

#### 4.1.2. The Influence of NXP on Ejector Performance

**Figure 9:**
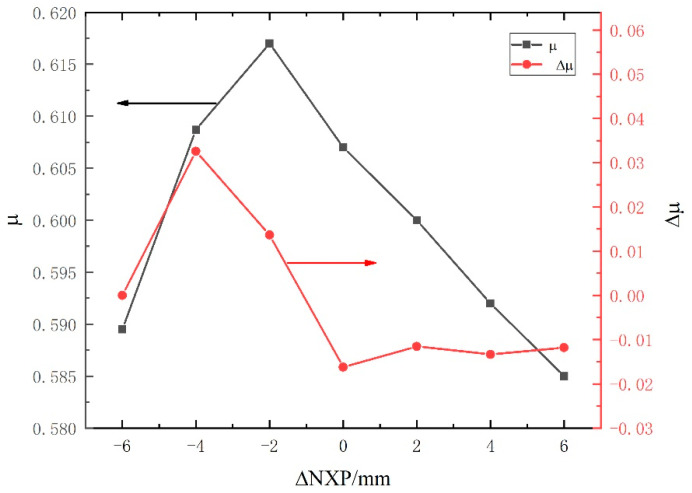
The effects of NXP on the entrainment ratio.

Figure 9 depicts the influences of NXP on the entrainment ratio. It should be pointed out that the ΔNXP is positive when the NXP increases, and it becomes negative when the NXP decreases. It can be concluded that the entrainment ratio increases initially and then decreases with the increase in NXP. The entrainment ratio decreases almost linearly with the increase in NXP when ΔNXP is positive, with a reduction rate of 1%. This means that an improper NXP will cause the rapid deterioration of the ejector performance. In addition, the entrainment ratio growth rate is positive when the ΔNXP is negative, and further increasing ΔNXP makes it becomes negative. As a result, the ejector has the best performance when ΔNXP = −2, with the maximum entrainment ratio having a maximum of 0.617. The improvement of the entrainment ratio is about 17.34% compared with the initial value.

**Figure 10:**
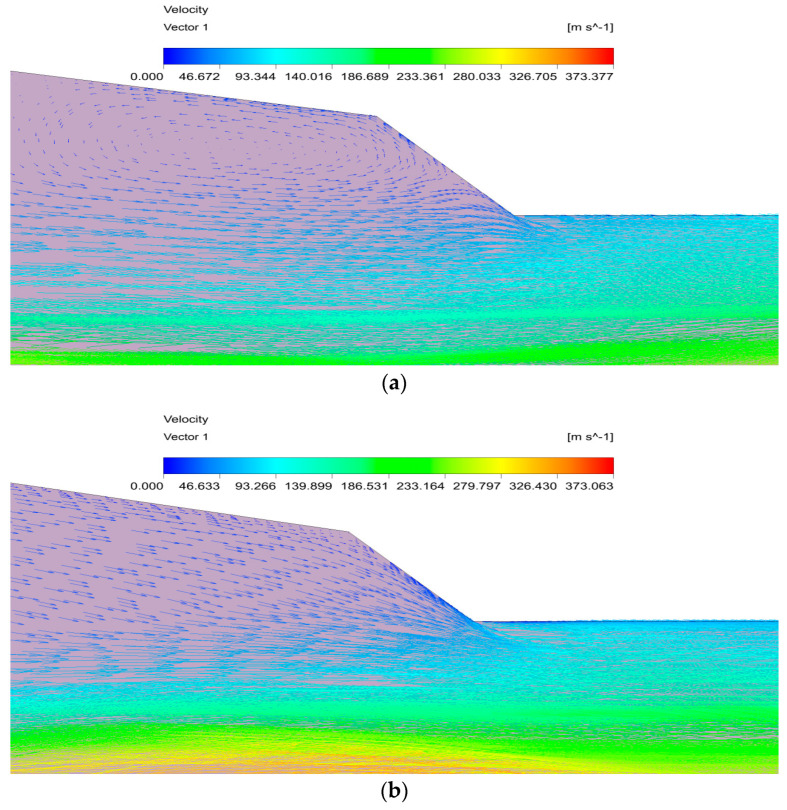
The velocity vectors in the suction chamber. (**a**) ΔNXP = 6 mm. (**b**) ΔNXP = −2 mm.

Figure 10 shows that there is an obvious vortex region in the suction chamber when ΔNXP = 6; however, this region disappears when ΔNXP = −2. As shown in Figure 10a, when the NXP is higher than the optimal value, the primary fluid becomes an over-expansion state, and the final section area of the motive fluid free flow beam is larger than the mixing chamber inlet area. As a result, the flow area is smaller for secondary fluid in the mixing chamber, resulting in the backflow of the secondary fluid. However, the pressure difference between the ejector inside and outside becomes larger due to the over-expansion of the motive fluid, resulting in the re-entrainment of the secondary fluid. This process causes the occurrence of the vortex region; under these circumstances, the energy loss inside the ejector increases, and the secondary fluid mass flow rate decreases, which results in the poor performance. The expansion degree of the working fluid is gradually appropriate with the decrease in NXP. The final section area of the motive fluid is exactly equal to that of the mixing chamber inlet when ΔNXP = −2. The entrained secondary fluid just enters the mixing chamber, and there is no backflow in the suction chamber. There is no extra energy loss inside the ejector at this time, so the ejector has the best performance.

**Figure 11:**
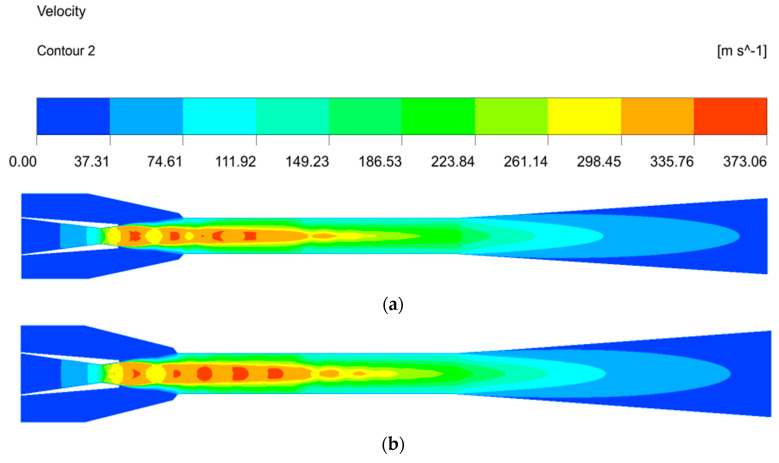
The effects of NXP on the velocity field. (**a**) ΔNXP = −2 mm. (**b**) ΔNXP = −6 mm.

Figure 11 indicates the velocity distribution under different NXPs. It can be seen that the velocity core of the primary fluid for ΔNXP = −2 mm is smaller than that for ΔNXP = −6 mm in the mixing chamber, which means that the velocity core increases with the decrease in NXP. When the NXP is less than the optimal value, the final section area of the motive fluid is smaller than that of the mixing chamber inlet, indicating that the motive fluid is under-expanded. As a result, the short NXP makes a smaller pressure difference between the primary and secondary fluid, causing a decrease in the secondary fluid mass flow rate. Moreover, the motive fluid continues to expand in the mixing chamber, resulting in a higher velocity core. At the same time, there is great irreversible loss inside the ejector due to the cyclic process of expansion–compression–expansion, resulting in the deterioration of the ejector performance. The velocity core decreases with the further increase in NXP, and the ejector has the best performance until the occurrence of an appropriate velocity core. In conclusion, the NXP mainly affects the primary fluid expansion—the motive fluid is over-expanded when it is longer than optimal; otherwise, the motive fluid is under-expanded.

### 4.2. Fluid Field Characteristic inside the Ejector with the Optimal Geometry Parameters

The better understanding of flow field characteristics is the basis of ejector performance optimization. As a result, this part analyzes the pressure field and velocity field characteristics inside the ejector under the optimal structural parameters to better understand the flow state and flow field characteristics of the fluid in the ejector.

#### 4.2.1. Pressure Distribution inside the Ejector

**Figure 12:**
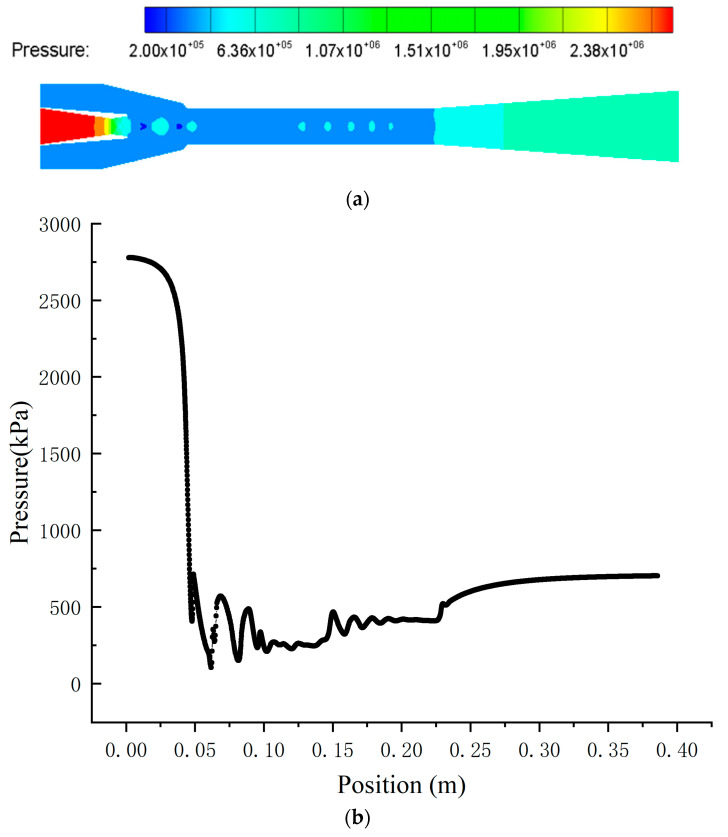
The pressure distribution inside the ejector. (**a**) The pressure contours inside the ejector. (**b**) The pressure distribution along the axis.

Figure 12 shows the pressure characteristics inside the ejector. A clear oscillating pressure phenomenon begins at the mixing chamber inlet and then disappears at the end of the mixing chamber. The fluid pressure decreases rapidly due to the decrease in the flow area and chocks in the nozzle throat when the motive fluid flows into the nozzle. The fluid further expands to the supersonic, creating a low-pressure region at the nozzle exit. Then, the secondary fluid is entrained into the ejector, and the low-pressure motive fluid undergoes the imperfect expansion process (also called the expansion–compression–expansion process) due to the pressure difference when the two fluids mix, resulting in the presentence of a shock train manifested by a series of oscillations of the static pressure along the axis of symmetry in the mixing chamber. Subsequently, the pressure inside the primary fluid is adjusted with that of the secondary fluid to attenuate the shocks until they disappear, and, finally, the mixing fluid reaches the same pressure at the end of the mixing chamber. The mixing fluid pressure increases and becomes subsonic after the second shock when the mixing fluid flows into the diffuser because of the increase in the flow area. Finally, the mixing fluid flows out of the ejector.

#### 4.2.2. Velocity Distribution inside the Ejector

**Figure 13:**
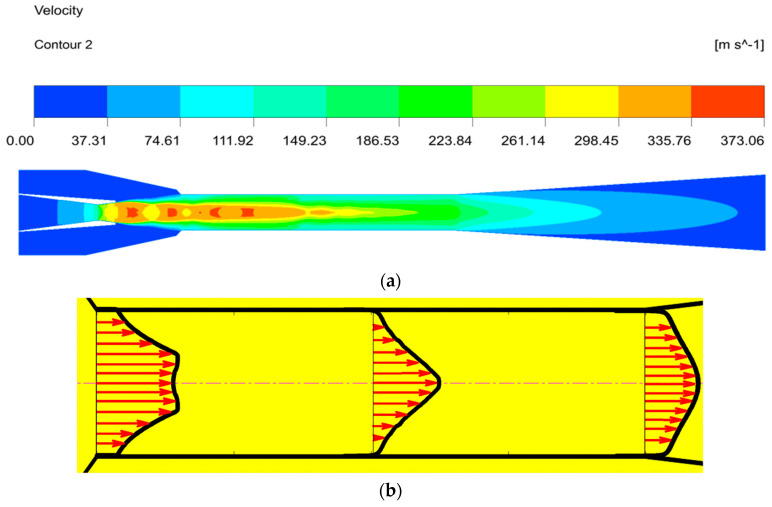
The velocity distribution inside the ejector. (**a**) The velocity contours inside the ejector. (**b**) The velocity vector inside the mixing chamber.

Figure 13 describes the velocity distribution inside the ejector. The velocity increases rapidly when the fluid flows through the convergent section of the nozzle, and the pressure potential energy of the motive fluid converts into the kinetic energy. The fluid velocity increases to supersonic as it flows through the nozzle divergent section, creating a low-pressure region at the nozzle exit, and then the secondary fluid is entrained, resulting in the formation of a shock wave, manifested by a series of oscillations of the velocity along the axis of symmetry in the mixing chamber. The velocity of the primary fluid decreases, while the secondary fluid velocity increases, and the mixing fluid velocity reaches about the same at the end of the mixing chamber. At this time, the shock trains disappear at the end of the mixing chamber after the momentum exchange process. Finally, the mixing fluid velocity further decreases to subsonic after the second shock in the diffuser, meaning that the kinetic energy transforms into the pressure potential energy. The fluid mixing characteristics in the mixing chamber, as an ejector essential component, influence the ejector performance. Figure 13b depicts the velocity vector distribution at the inlet, outlet and half of the mixing chamber. It can be seen that the velocity of the secondary fluid is very small, the motive fluid has a high-velocity core and there is a great velocity difference, which indicates that the mixing of the two fluids is not sufficient at the mixing chamber inlet. The velocity of the entrained fluid gradually increases as the mixing progresses; at the same, the motive fluid velocity decreases and the velocity core disappears, meaning that the kinetic energy of the two fluids is being exchanged at the half of the mixing chamber. The kinetic energy exchange of the two streams is sufficient, the velocity of the two fluids tends to be the same and the mixing is completed at the end of the mixing chamber. In addition, there is no backflow in the whole mixing chamber, which also indicates that the ejector is well designed.

## 5. Conclusions

This paper proposes a thorough ejector design method for R1234yf based on the gas dynamic methods and optimizes the ejector geometrical parameters to improve the R1234yf ejector refrigeration system performance through control variable optimization algorithms. Based on the verified numerical simulation model, the following results are obtained:

(1) Indicate the coupling laws between the area ratio and the ejector performance. When the area ratio increases, the entrainment ratio increases initially and then decreases; the recirculation area in the mixing chamber first decreases and then increases with the increase in the area ratio. There is no recirculation inside the ejector when the area ratio is 5.28, and the shock wave disappears exactly at the end of the mixing chamber. At this time, the entrained secondary fluid just passes through the mixing chamber without additional energy loss, and the maximum entrainment ratio is 0.602.
(2) Reveal the relationships between NXP and ejector performance. The entrainment ratio first increases and then decreases with the increase in NXP, and the change in NXP directly affects the expansion state of the motive fluid. When ΔNXP is −2 mm, the expansion degree of the motive fluid is appropriate, and the final section area of the motive fluid beam is exactly equal to that of the mixing chamber inlet. There is no backflow phenomenon in the suction chamber, and the fluid streamline is clear, with the maximum entrainment ratio being 0.617.
(3) Obtain the optimal entrainment ratio through the control variable optimization algorithms. The entrainment ratio increases by 17.34% compared with the initial value.

In conclusion, this paper provides a design method for the R1234yf ejector based on the aerodynamic method and optimizes the ejector geometrical parameters through control variable optimization algorithms. In addition, the coupling laws between the area ratio, the nozzle exit position and the ejector performance are obtained, which can promote the development of the ERS with R1234yf.

## Figures and Tables

**Table 1:** The main ejector geometrical dimensions.

Parameters	Value (mm)
Diameter of the nozzle inlet	17.4
Diameter of the nozzle throat	7.4
Diameter of the nozzle outlet	9.6
Diameter of the mixing chamber	20
Diameter of the diffuser outlet	39.6
Length of the nozzle convergence section	40.8
Length of the nozzle divergence section	9
Length of the mixing chamber	140
Length of the diffuser	160

**Table 2:** Grid independence verification at the mixing chamber inlet.

Mesh Number	Pressure (Pa)	Error (%)	Velocity (m/s)	Error (%)
94,258	498,590.5		290.6628	
141,489	503,297.0	0.944	290.1832	−0.165
219,708	503,000.0	−0.059	290.12048	−0.02

**Table 3:** The comparisons between the simulation results and the experimental results.

Generator Temperature (°C)	Evaporator Temperature (°C)	Condenser Temperature (°C)	Entrainment Ratio		Errors (%)
			Experimental Results	Simulation Results	
95	8	31.3	0.4377	0.3854	11.95
84	12	28.9	0.6350	0.7247	14.13

## Data Availability

Not applicable.

## Nomenclature

a	critical velocity (m/s)	c	the mixing chamber outlet
d	diameter (mm)	np	the critical state
f	area (m^2^)	p	the primary fluid
l	length (mm)	s	the secondary fluid
m	mass flow rate (kg/s)	*Abbreviations*	
P	pressure (Pa)	AR	area ratio
PR	pressure ratio	CFD	computational fluid dynamics
*Greek letters*		ERS	ejector refrigeration system
μ	entrainment ratio	NXP	nozzle exit position
φ	velocity coefficient	RANS	Reynolds-Averaged Navier–Stokes simulation
Π	relative pressure		
ρ	density (kg m^−3^)		
